# Access and Fluoroscopy Time Difference in Patients Undergoing Prone Percutaneous Nephrolithotomy (PCNL) With Ureteric Catheter Placement in Supine Versus Lithotomy Position

**DOI:** 10.7759/cureus.26220

**Published:** 2022-06-22

**Authors:** Kaleem K Mirani, M Hammad Ather, Zehra Kazmi, Wajahat Aziz

**Affiliations:** 1 Urology, Aga Khan University, Karachi, PAK; 2 Urology and Surgery, Aga Khan University, Karachi, PAK

**Keywords:** ureteral catheter, access technique, operative time, fluoroscopy time, pcnl

## Abstract

Objective: To compare the operative and fluoroscopy time in two different methods of ureteral stent insertion before prone percutaneous nephrolithotomy (PCNL).

Materials and method: Over 12 months, 124 patients with urolithiasis who went through prone PCNL were included in our study. All the patients had kidney stones and were divided into two groups based on the method of ureteral catheter insertion. This was done with the help of flexible or rigid cystoscopy in to group A and group B, respectively. Both groups had an equal number of patients, i.e., 62. The main outcome variables assessed were access time (from intubation to placement of access sheath) and fluoroscopy time during access.

Results: The categorical variables (age, gender, site of the stone) between the two groups were comparable. The access time in groups A and B were 48±4.30 and 77±10 minutes, respectively. The fluoroscopy times in groups A and B were 52±14.63 and 116±47.77 seconds, respectively. A statistically significant difference (p-value < 0.05) was observed for both the access and fluoroscopy time. None of the patients in either of the groups had a misplaced ureteral catheter requiring repositioning.

Conclusion: Flexible cystoscope-assisted insertion of ureteral catheter prior to PCNL significantly reduces operative time, fluoroscopy time, and consequently radiation exposure during PCNL.

## Introduction

One of the most significant steps in percutaneous nephrolithotomy (PCNL) is to get safe access to the pelvicalyceal system [1]. An open-end (ureteric catheter) is often placed to create distension in the collection system to ease access into the calyx/calyces of interest. A ureteric catheter is also helpful to flush back any fragment that has dropped into the proximal ureter and by partially occluding the ureteropelvic junction to prevent the downward migration of stone fragments. It can also be used for retrograde guide wire insertion and postoperative drainage of the kidney.

Retrograde access with an open-end ureteric catheter is often obtained by rigid cystoscopy in a lithotomy position. In prone PCNL, it is a time-consuming and cumbersome procedure as it involves making lithotomy position following general anesthesia, preparation and draping, and then repositioning and shifting the patient on the gurney or patient transfer trolley, followed by replacement on the operating room table in the prone position. Flexible cystoscopy for ureteric catheterization and retrograde studies under local anesthetic is a commonly performed procedure [2,3]. An alternate method is to place an open-end catheter in the supine position using flexible scope following intubation on the gurney.

Fluoroscopy, ultrasonography (US) and computed tomography (CT) are all validated and essential tools for performing a successful PCNL [4]. Despite recent advancements, fluoroscopy remains the surgeon’s choice due to reasons of familiarity and ease of use [5]. Fluoroscopy though an indispensable tool for endourologists, is associated with significant radiation exposure [6]. Various modifications have been described to reduce radiation exposure during PCNL [7,8] and overall radiation exposure in patients with urolithiasis [9]. Amongst all the urological procedures, PCNL has the highest dose and time of radiation exposure [6,10].

As per the established principle of as low as reasonably achievable (ALARA), one of the key disciplines of advancement in the contemporary treatment of urolithiasis is to reduce radiation exposure [11]. Fluoroscopy time (FT) is an important factor during PCNL that needs to be controlled to minimize radiation exposure [12]. The technique of ureteric catheter placement using flexible cystoscopy may lessen the access time and fluoroscopy radiation dose.

We aim to compare the access time and fluoroscopy time used in lithotomy versus supine position for ureteric access before PCNL.

## Materials and methods

Research methodology

Our study was performed in the urology department of the tertiary care hospital. This is a cross-sectional study and for which institutional review board endorsement (approval no.2021-5381-15388) was taken. The sample size was calculated using the OpenEpi program (version 3), with a power of 80%, a two-sided confidence interval of 95%, an anticipated mean difference of 1.02 of access time in the two groups, and a standard deviation of 1.76 and 2.25, respectively [13]. The study group comprised 124 adult patients presenting to our center with the diagnosis of renal calculi requiring PCNL. Data were collected over one year via a non-probability consecutive sampling technique. Access time is defined as the time from anesthesia induction to successful placement of access sheath. Fluoroscopy time was calculated by C-arm fluoroscope from the start of the procedure till successful placement of access sheath. Procedure time is defined as the time from placement of access sheath to dressing after nephrostomy placement.

The study participants were divided into two groups based on the method of ureteric catheter placement i.e., via flexible cystoscopy in the supine position (group A, n=62), and via rigid cystoscopy in the lithotomy position (group B, n=62). Demographic data collected for each patient included age, gender, BMI, and site of stone disease. Patients with incomplete medical records, a history of having had any renal procedure, bilateral stones, co-existing ureteric or bladder pathology, hematologic disorders, and those requiring ancillary procedures or more than one puncture, were excluded from the study. 

All the patients underwent laboratory investigations prior to the procedure and this workup included serum hemoglobin, creatinine, clotting workup, and urine tests including urine CS. These reports were reviewed in clinics and abnormal values were addressed in detail before admission to prevent any last-minute changes in planned surgery and inconvenience to the patient. Patients with positive urine cultures were treated with antibiotics as per sensitivity before the procedure. The radiologic assessment was performed with a non-contrast CT scan of the kidneys, ureters, and bladder (KUB). Stone dimensions were calculated by an experienced radiologist via system-generated software but are not recorded in our study and no comparison was made between the two groups. To maintain the anonymity of patients, all patients were given a code linked with their hospital record number. Original data with hospital record number and study code was put under lock and key and within the approach of the principal researcher only. 

Operative technique

The procedure was performed by one of the three urologists with at least 60 cases performed by each urologist before the procedure in the study population. In group A, the patient was anesthetized on the stretcher which was used to shift the patient from the pre-operative area to the operating room, and a 5F ureteral catheter was inserted under direct vision through the flexible cystoscope in the supine position. As it is difficult to access the external urethral opening in the supine position in female patients, the frog-leg position was used for ureteral catheter insertion. In this group of patients, fluoroscopy was not utilized in any of the patients during ureteral access. As fluoroscopy was not used, urine drainage was used as a surrogate marker for position confirmation. Foley's catheter was passed and ureteral stent internalized to avoid dislodgement during position change and both were placed in the sterile bag. Then, the patient was shifted from the gurney to the operating table for a definitive procedure.

In contrast, group B patients were shifted from the gurney to the operating table, anesthetized, and placed in the lithotomy position. Rigid cystoscopy was done and a ureteral catheter was placed. Fluoroscopy was used to guide and confirm the position of the ureteral catheter, as well as for retrograde pyelography. The ureteral catheter and the Foley catheter were similarly secured in a sterile bag. After this, the patients were made supine and shifted to the gurney to make a prone position. The patients were shifted once again to an operating table in the prone position at this time. 

The access time encompassed four sub-domains: (1) Positioning the patient for cystoscopy after intubation, (2) cystoscopy-guided ureteral catheter placement, (3) prone positioning for the definitive procedure, and (4) puncture followed by tract dilatation and successful placement of 22 to 26 Fr Amplatz access sheath. After stone disintegration and retrieval, a 14F nephrostomy tube was inserted at the end of the procedure in all cases. The access time was calculated from induction of anesthesia to successful placement of access sheath, and procedure time began from successful placement of access sheath to dressing after placement of nephrostomy at the end of the procedure. General patient data and other pertinent procedure-related findings were recorded on a proforma designed for this purpose.

Data analysis

Data analysis was performed using Statistical Package for the Social Sciences (SPSS) v26 (IBM Corp., Armonk, NY, USA). Descriptive statistics have been reported as frequency and percentages for categorical variables. Mean ± standard deviation has been reported as appropriate. Quantitative variables between the two groups were compared using the paired t-test, p-value of <0.05 were taken to be statistically significant.

## Results

A total of 124 adult patients who underwent PCNL for the first time, were included in the study. Group A had 39 male and 23 female patients. Group B comprised 45 men and 17 women. The Chi-square test demonstrated the similarity in gender between the two groups (p-value=0.923). The average age was comparable between the two groups: 45.04±15.27 in group A, and 42.9±15.07 in group B (p-value=0.22) (see Table 1). Thirty patients in group A underwent left-sided PCNL, and 32 were operated on the right side. In group B, left-sided stone surgery was performed in 28 patients, while 34 underwent right-sided PCNL. The differenconcerningtwo groups with respect to the site was not significant as demonstrated by the Fischer exact test (p-value=0.8573). Successful ureteric access was obtained at the first attempt in both groups. Body mass index (BMI) was recorded for all individuals. A statistically significant difference in BMI was found between the two groups. The average BMI in group A was 25.5±3.75, which was less than the average BMI noted in Group B (26.95±4.76), and had a p-value of 0.03. We compared the operative time, and fluoroscopy time during access. The difference between the two groups was statistically significant for both categories, with a p-value of <0.005 as shown in Table 1.

**Table 1:** Demographic data and outcome variables in the study group (A) and control group (B) BMI: Body mass index

Variable	Group A (Mean ±SD)	Group B (Mean ±SD)	p-value
Total patients	62	62	-
Male	39	45	0.923
Female	23	17	
Age (years)	45.04±15.27	42.9±15.07	0.22
BMI (kg/m^2^)	25.5±3.75	26.95±4.76	0.03
Side	Left: 30, Right: 32	Left:28, Right: 34	0.8573
Access time (in minutes)	48±4.30	77±10.02	<0.05
Total procedure time (in minutes)	51±32.41	54±26.85	0.462
Fluoroscopy time (in seconds)	52±14.63	116±47.77	<0.05

**Figure 1:**
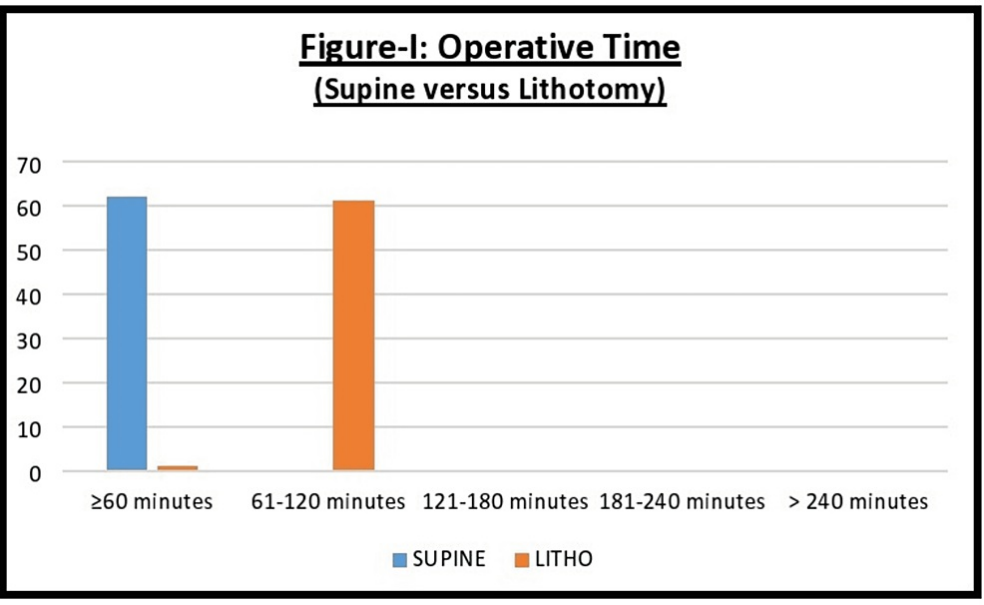
Operative time difference between the two groups, i.e., supine and lithotomy LITHO: Lithotomy

Figure 1 demonstrates the operative time i.e., the time from anesthesia induction to successful placement of access sheath. The mean access time in group A was 48±4.30 minutes, and 77±10.02 minutes in group B, making it statistically significant.

**Figure 2:**
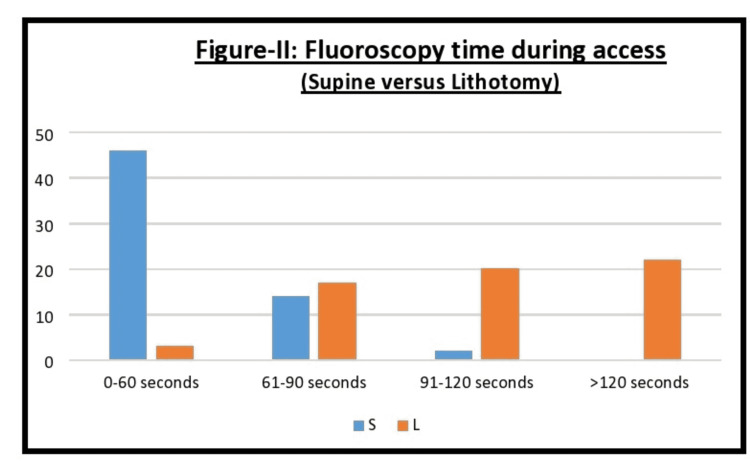
Fluoroscopy time during access difference between the two groups i.e., supine versus lithotomy S: Supine, L: Lithotomy

For a better comparison of fluoroscopic use in both groups, we subdivided each group into categories based on seconds: use of fluoro for 0-60 seconds, 61 to 90 seconds, 91 to 120 seconds, or more than 120 seconds. Similar differences were observed while comparing the fluoroscopy time during the access technique as shown in Figure 2. The mean fluoroscopy time for gaining access in group A was 52±14.63. In 46 cases, the fluoroscopy exposure was from 0 to 60 seconds, in four it was 61 to 90 seconds, and in two cases it was from 91 to 120 seconds. In contrast, group B's average fluoroscopy time was 116±47.77. In three cases it was from 0 to 60 seconds, in 17 it was for 61 to 90 seconds, in 20 it lasted for 91 to 120 seconds, and 22 cases required fluoroscopy use for >120 seconds (Figure 2). The difference in fluoroscopy use for gaining access between the two groups was statistically significant (p<0.05). We also compared the total procedure time between the two groups. The mean time in group A was 51±26.8 minutes, which when compared to group B's 54±32.41 (p=0.46), was not statistically significant.

## Discussion

In the Asian population, the incidence of urolithiasis is between 1% to 19.1% of the population [14]. In Pakistan, the prevalence of urolithiasis is 16% with an incidence varying from 0.2 to 200 per 100,000 of the population [15]. In the last two decades, endoscopic urology has revolutionzed the field of urology and a major part of stone surgery burden has shifted from old open surgery method to the endoscopic method. This pragmatic change has not only influenced the developed world but also the low and middle income countries of Asia, too [16]. Percutaneous nephrolithotomy was introduced in 1976; subsequent technologic developments and refinement in endourologic techniques have made PCNL the management option of choice for large renal stones and/or stones that are resistant to shock wave lithotripsy (SWL) or ureteroscopy [17]. Although prone PCNL has been in vogue for decades, recent advances have introduced a supine position for PCNL and different modifications have also made it possible. These recent advances were made due to limitations of prone PCNL and these limitations include the need to change the position more than once, difficulty in performing endoscopic combined intrarenal surgery (ECIRS), and endotracheal tube compression due to increased pressure, and prolonged surgery time. But despite these limitations, prone PCNL is the most common form of PCNL worldwide [18].

One of the major advantages of the supine PCNL over prone PCNL is that it obviates the need for patient repositioning and allows combined ureteroscopic procedures. However, the traditional prone position continues to be widely practiced [19]. In our center, over the past 30 years, PCNL is performed in the prone position. De la Rosette et al. studied the different forms of PCNL in complex stones in a retrospective manner and found that prone PCNL is a better position for complex stones with significant improvement in success [17]. Longer operating time is one of the determinants of complications during PCNL [20]. While there are a variety of studies analyzing the many aspects that affect access time, most have not taken into consideration the time taken to prepare for the procedure [21]. In our study, we performed all the PCNL cases in the prone position. We looked at one dependable way of minimizing the duration of the pre-PCNL phase by using the flexible cystoscope for ureteric access. We then compared the outcome with the rigid cystoscope. In this study, a senior urology resident experienced in the usage of both flexible and rigid instruments performed the initial placement of the ureteric catheter. The main advantage of using a flexible cystoscope for ureteric catheter insertion before PCNL is its practicality, which is beneficial for patients, operating room staff, and the anesthesia team. Besides this, the supine position is more suitable for patients with lower limb contracture or recent orthopedic surgery which preclude the lithotomy position. Also, the lithotomy positioning of a patient for rigid cystoscopy is cumbersome for both the patient as well as the operating team [3]. The need for repositioning an intubated patient not only requires manpower but is also associated with life-threatening complications [22]. Wu et al. described awake endotracheal intubation and this could help the patient to self-position and decrease the workload and need for manpower during PCNL [23].

Fluoroscopy, US, and CT are commonly approved modalities used for radiologic guidance especially while performing the first step of the PCNL, which is percutaneous access. Tepeler et al. emphasized the use of air pyelography to allow rapid identification of the appropriate access site, unobscured views of the stone, and decreased radiation exposure [24]. Reduction in radiation exposure both for the patient and staff is important in the management of urolithiasis. Distance, shielding, and time are critical factors that determine radiation exposure. Active personnel dosimeters are absolutely essential for achieving the ALARA principle [25].

The maximum yearly whole-body exposure as proposed by the National Commission on Radiation Protection is 5 rem. This limit is not minimal and a surgeon may carry out up to 400 PCNLs in a year without the extra risk of radiation-induced morbidity [26]. Keeping a check on radiation exposure is essential and there should be methods to decrease radiation exposure so that more PCNL surgeries can be performed without risk. This need compelled us to investigate the reasons for radiation exposure and how we can minimise it. Fluoroscopy time is an objective surrogate marker of radiation exposure and we observed it in our study. We also studied the preparation period before the definitive procedure i.e., the time from intubation to positioning for cystoscopy, ureteral catheter insertion, and the final position for surgery. We found that ureteral stenting via flexible cystoscope is significantly less time-consuming than rigid cystoscopy (48±4.30 vs 77±10.02, p<0.05).

Like all other studies, our study also has a few limitations. The cost effectiveness and pain factor in the two different methods of ureteral catheter insertion were not calculated. It is postulated that flexible cystoscope causes less harm to mucosal tissue in comparison to rigid cystoscope but we have not studied it. The difference in outcome with respect to stone size, type, and degree of hydronephrosis was not evaluated.

## Conclusions

Two methods of ureteric catheter insertion are used during PCNL i.e., supine and the lithotomy position. In the lithotomy method, rigid cystoscopy is used and the patient is made to take two different positions. This results in extra operating time and is counter-productive. It also increases the radiation dose. In contrast, the supine approach utilizes flexible cystoscopy and there is no use of fluoroscopy, which saves time, reduces radiation exposure, and is cost-effective. Flexible cystoscopy-aided placement of ureteric catheter provides the added advantage of significantly less fluoroscopy exposure for the patient as well as the operating team. Larger multi-institutional studies are needed to explore further the advantages of this approach.
